# Legal Notes for Institutional Workers

**Published:** 1912-08-17

**Authors:** 


### LEGAL NOTES FOR INSTITUTIONAL WORKERS

**(The Editor will be pleased to answer Legal queries in this column.)**

### Contracts with Hospital Authorities.

Where there is a contract for the erection of, or to supply goods to, a hospital, it is not always easy to ascertain who is responsible for payment. If the hospital governors are a body corporate, no great difficulty arises, because the general law as to contracts with corporations is then applicable; but in the case of hospitals which are not incorporated different considerations arise, as there is no legal entity or person representing it. It appears that an agreement made by an individual or committee is primarily binding on such individual or committee, who must look to the funds of the hospital for their indemnity. Evidence rebutting this primary liability may, however, be produced to show that the person employed or the tradesman who supplied goods agreed to trust to payment out of the available funds of the hospital itself and to no other source.

### Whether Subscribers or Officials Liable.

Again, the mere fact that a hospital has a large body of subscribers gives to the contractor no real security. The subscribers and donors to a hospital do not by virtue of their subscriptions and donations incur any personal liability for work done or goods supplied by the direction of the managing committee; for members of a committee have no authority to pledge the personal credit of those who have supplied the fund for carrying on the hospital, and their right of indemnity is limited to such fund (see Murray's *Law of Hospitals*, pp. 33, 34). A hospital official who attempts to bind the hospital employing him, whether it be incorporated or unincorporated, by an agreement which is in excess of the powers conferred by the hospital rules, is personally liable without any right to recoupment out of the hospital funds for any loss which may be incurred.

